# Lean Mass Improvement from Nutrition Education and Protein Supplementation among Rural Indian Women Living with HIV/AIDS: Results from Cluster Randomized Factorial Trial at 18-Month Follow-Up

**DOI:** 10.3390/nu14010179

**Published:** 2021-12-30

**Authors:** Catherine L. Carpenter, Kavita Kapur, Padma Ramakrishna, Suresh Pamujula, Kartik Yadav, Jennifer E. Giovanni, Olivia Julian, Maria L. Ekstrand, Sanjeev Sinha, Adeline M. Nyamathi

**Affiliations:** 1 Center for Human Nutrition, University of California, Los Angeles, CA 90024, USA; jegiovanni@gmail.com; 2 Whitefield, Bangalore 560066, India; kavitakapur@hotmail.com; 3 People’s Health Society, Nellore 524137, India; rkpadmaashaproject@gmail.com (P.R.); suripamujula@gmail.com (S.P.); 4 Sue & Bill Gross School of Nursing, University of California, Irvine, CA 92697, USA; k.yadav@uci.edu (K.Y.); anyamath@hs.uci.edu (A.M.N.); 5 College of Medicine, Drexel University, Philadelphia, PA 19129, USA; ojulian@ucla.edu; 6 Center for AIDS Prevention, Department of Medicine, University of California, San Francisco, CA 94158, USA; Maria.Ekstrand@ucsf.edu; 7 All India Institute of Medical Sciences (AIIMS), New Delhi 110029, India; drsanjeevsinha@gmail.com

**Keywords:** lean mass, HIV/AIDS, protein, nutrition, cluster-randomized trial

## Abstract

Loss of lean muscle mass impairs immunity and increases mortality risk among individuals with HIV/AIDS. We evaluated the relative contributions of protein supplementation and nutrition education on body composition among 600 women living with HIV/AIDS in rural Andhra Pradesh, India. We conducted a cluster randomized controlled 2 × 2 factorial trial lasting six months with follow up at twelve and eighteen months. Interventions occurred in the Nellore and Prakasam regions of Andhra Pradesh by trained village women, ASHA (Accredited Social Health Activists), and included: (1) the usual supportive care from ASHA (UC); (2) UC plus nutrition education (NE); (3) UC plus nutritional protein supplementation (NS); (4) combined UC plus NE plus NS. A Bioimpedance Analyzer Model 310e measured body composition. SAS 9.4 analyzed all data. Mixed models using repeated measures evaluated lean mass change from baseline as primary and fat weight and total weight as secondary outcomes. Lean mass change was significantly associated with NS (*p* = 0.0001), NE (*p* = 0.0001), and combined NS plus NE (*p* = 0.0001), with similar associations for secondary outcomes. Stronger associations for total weight were observed with greater ART adherence. Nutritional interventions may improve physiologic response to HIV. Significant increases in lean mass resulted from independent and combined protein supplementation and nutrition education.

## 1. Introduction

A healthy immune response to infectious disease is dependent upon adequate nutrition. Calorie depletion, lack of nutrients, and diminished absorption can lead to immune deficiency; thereby increasing susceptibility to infectious diseases, worsening prognosis, and increasing risk of mortality [1].

People living with HIV/AIDS are at a high risk of nutritional deficiency [2]. Poor nutritional status prior to disease onset promotes reduced lymphocyte numbers and weakens the immune response resulting in a more rapid rate of infection by the HIV virus [3,4]. Once infected, stimulation of the immune response increases demand for critical nutrients associated with enzymatic reactions and substrates, and this excess strain on the immune system can lead to nutritional deficiency [3]. If these deficiencies occur at the start of anti-retroviral therapy (ART), the risk of mortality is increased independent of immune status [5,6,7]. Finally, nutrient deficiencies can, through weakening of the immune response, also increase susceptibility to infection from opportunistic diseases [2].

Apart from calorie depletion and generalized malnutrition, particular nutrient deficiencies have been linked to worsening prognosis from HIV/AIDS [7,8]. While results from studies are mixed, there is some evidence that supplementation with protein [8,9]; carotenoids including vitamin A [10]; iron in combination with other micronutrients [11]; zinc [12]; and selenium [13] have resulted in improved CD4 counts, prolonged survival, and increases in muscle mass and body composition [8,9,10,11,12,13].

Several physiologic signs of nutrient depletion have been linked to poorer prognosis among individuals living with HIV/AIDS. The pathogenesis of HIV and resulting metabolic demands, particularly in regions of endemic malnutrition, produces progressive weight loss from anorexia, muscle mass reduction from catabolism, stressors on immune response due to secondary infections, and gastrointestinal disturbances impairing nutrient absorption [14]. In particular, loss of lean muscle mass has been associated with a more rapid decline of immune function and increased risk of mortality among individuals suffering from HIV [15].

We assessed the relative contributions of nutritional protein supplementation and nutrition education on body composition outcomes by conducting a cluster-randomized 2 × 2 factorial trial among 600 women living with HIV/AIDS in the rural region of Andhra Pradesh, India. Our primary outcome was change in lean mass, with secondary outcomes represented by changes in fat mass and weight. Our trial consisted of four intervention conditions arranged in a factorial design [16]. Our usual care control intervention was implemented by a nurse-led healthcare AIDS Self-Management program that included community-based supportive care delivered by trained lay village women, ASHA (Accredited Social Health Activists) [9,17,18]. The three test conditions consisted of differing levels of nutritional supplementation or nutritional education. The four interventions included: (1) supportive care provided by ASHA; (2) ASHA support plus nutrition education; (3) ASHA support plus nutrition supplementation; and, (4) ASHA support plus nutrition education plus nutrition supplementation.

## 2. Materials and Methods

### 2.1. Design

**Figure 1:**
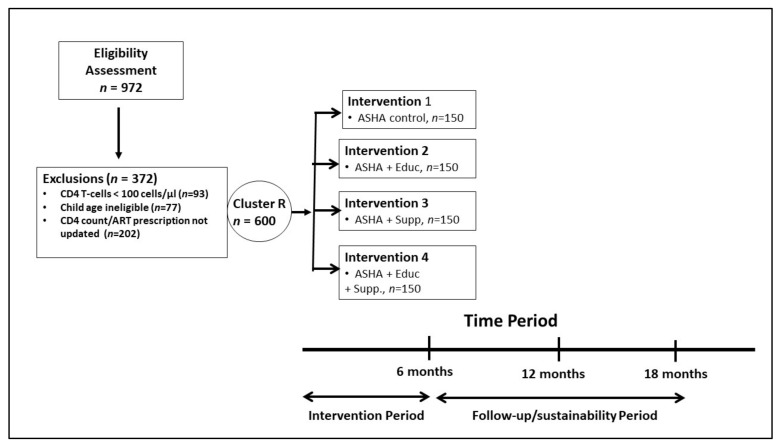
Design of ASHA-Nutrition Study.

We conducted a prospective cluster randomized controlled 2 × 2 factorial design to assess the impact of ASHA-supported interventions and selected nutritional components on key disease-related outcomes in HIV-positive WLA (women living with HIV/AIDS) in India (*n* = 600) over a six-month period. Follow up assessments occurred at six, twelve, and eighteen months post enrollment (see Figure 1). Written informed consent was obtained from all participants. Study protocol was approved by Human Subjects Protection Committees at the U.S. based Universities of California at Los Angeles, at Irvine, and at San Francisco in the United States and the Ministry of Health in India. Baseline data collection was staggered over a two-year period (May 2014–November 2016), enrolling 100 women at a time (25 per condition) in six rounds.

### 2.2. Sample and Setting

WLA between the ages of 18–50 with a verified HIV-positive diagnosis, anti-retroviral therapy (ART)-prescription for the last three months, living with a child aged three to eight, and receiving care in a selected primary care centers (PHCs) located in the Nellore and Prakasam districts of Andhra Pradesh (AP) were recruited for participation. We conducted earlier pilot research in the same region and were familiar with the geography, villages, location of PHCs and population groups who lived there [9,17,18]. There were 16 PHCs (Primary Care Centers) that were grouped into four regional clusters. The clusters were created so they contained villages that were close to each other and yet distant from villages contained in other clusters. In addition, each cluster was served by a different PHC, so chance encounters at the PHC by participants from different clusters would not occur. This design was implemented rather than randomizing each of the individual 16 PHCs into one of the four arms due to concern for contamination of intervention conditions. Villages contained in the cluster were generally small and women in one village often knew other women in the same village and neighboring villages contained within the same cluster. We arranged clusters according to geographic proximity and PHC containment areas in order to increase feasibility and promote intervention fidelity. Once the clusters were identified, we allocated interventions to clusters within the overall region in the Nellore and Prakasam districts using a randomization procedure. Our study site office was located in Kovur, a village adjacent to Nellore, and centrally located to the four intervention sites.

Exclusion criteria included CD4 T-cell levels < 100 cells/mm^3^ and participation in previous ASHA-interventions led by our team. We posted recruitment flyers in the selected PHCs; interested women met with trained research staff in a private location to receive study information, provide informed consent (if interested) and complete an eligibility screener. Eligible participants completed a second informed consent.

We verified HIV diagnoses by checking participants’ hospital-generated ART card. Testing of CD4+ T helper cell count occurred during screening. In total, we screened 974 WLA with 600 recruited (see Figure 1). Ineligibility resulted from ART prescription not updated in the past three months (51%), CD4 T-cell levels < 100 cells/mm^3^ (31%), and child age not eligible (18%).

WLA received total incentives ranging from Rupees 9036 to 9562 (USD $172 to USD $182) to cover transportation costs, loss of pay for the time of enrollment, or childcare as needed.

### 2.3. Asha Selection and Training

In our previous pilot intervention trial, we evaluated the use of ASHA to execute the supportive care portion of the intervention [9]. The project director (PD) selected lay village women to become ASHA. ASHA eligibility included those aged 20–50; educated at least to the 8th grade level; were interested in caring for women and children with HIV/AIDS; had a history of community service; were living either in study site villages or in close proximity, and belonged to similar castes as the WLA.

The Principal Investigators (PIs) and Project Director (PD) trained, in total, 16 ASHAs. ASHA training included education about the study protocol; the needs of WLA; the biology of HIV/AIDS disease progression; adherence to ART; coping with HIV illness symptoms; maintaining participant confidentiality; and preserving intervention fidelity. After training, each intervention condition received four ASHAs, who worked within their assigned intervention site throughout the study. Conduct of quality assurance assessments on a quarterly basis functioned to assure compliance to study protocols. ASHAs, employed full time, were paid a monthly stipend of Rs.3450 (US $54); commensurate with typical salaries from the government for providing reproductive health-related services to women in these communities. 

### 2.4. Description of Sampling and Asha Nutrition Intervention Programs

We identified four high prevalence geographic sites within the rural district of Nellore, in the state of Andhra Pradesh [9], and randomly allocated these sites to one of four intervention conditions. Cluster randomization was the chosen sampling scheme because randomization of individuals across villages was not feasible due to transportation challenges and the potential for loss of intervention fidelity [19].

A detailed description of the four interventions are presented in earlier publications of the study results [20,21] and are summarized below.

Program 1 (ASHA support only) served as the control group. ASHA-support consisted of ASHAs visiting six to seven WLA weekly on a 1:1 basis, monitoring barriers to and recording of ART adherence, accompanying WLA to hospital and clinic visits, and assisting with removal of barriers to accessing health care including explaining prescribed treatments. Participants also learned to seek support from ASHA and physicians, were taught about HIV/AIDS disease progression and treatments and maintaining healthy routines. Additional modules included care-giving skills and promotion of positive psychological states. 

Program 2 (ASHA support + nutrition education) sessions provided education about consuming foods rich in nutrients (Vitamin A, iron, selenium, and zinc) whose deficiencies are linked to immune suppression or worsening of HIV/AIDS [8,9,10,11,12,13]. The nutrition investigator, India-based dietician and project manager, using dietary guidelines for Indians, suggested choices of inexpensive foods indigenous to the region that could protectively strengthen immune responses. These foods were inexpensive and easy to find in the four study sites. As an example, the nutrition education program recommended daily consumption of the ‘Drumstick Vegetable’ (*Moringa oleifera*) and leaves from the Drumstick Vegetable tree, which grows wild in the region, are easily obtainable without cost, and are rich in protein, Vitamin A and iron. The nutrition classes, designed to educate WLA on maintaining nutritional value within cultural preferences, consisted of, in addition to recommendations, cooking classes and recipe exchanges. WLA were instructed where to purchase inexpensive food and how to monitor food intake. The Nutrition Investigator taught the nutrition education program to the Project Interviewers who then delivered the education program to the participants.

Program 3 (ASHA support + food supplementation) provided monthly food supplements that consisted of high protein dals (lentils). WLA received three packs of food (i.e., 3kg each of black gram (Urad dal) and Pigeon peas (Toor dal) on a monthly basis when WLA visited the study office to attend group meetings. Food for the entire family was provided to ensure the women themselves received adequate amounts. Per 100 g, the black gram consisted of 347 calories, 24 g of protein, 1.4 g of fat and 59.6 g of total carbohydrates. The Toor dal per 100 g consisted of 335 calories, 22.3 g of protein, fat (1.7 g) and 57.6 g of carbohydrate. The amount of food was calibrated to represent an addition of 500 kcal (bowl-sized serving) to the daily diets of each woman.

All intervention nourishment was purchased from a local grain provider in Kovur. Each nutritional supplement package was consistently mixed in prescribed amounts, weighed, and allotted to each participant.

Program 4 (ASHA support + nutrition education + nutrition supplementation) combined all elements from Programs 1, 2 and 3.

### 2.5. Assessment Measures

We utilized validated instruments to assess physical, mental, social and psychological health at baseline and at the end of the intervention period. Further detailed information about assessment measures is available in our earlier publications [20,21], and summarized as follows. Instruments were adopted from studies of persons living with HIV (PLWH) in rural [17,18] or urban [22,23,24,25] settings in southern India. Instruments were translated into Telugu, the language used in our study site region.

Behavioral and psychosocial measures represented measurements of psychological and social factors that included: Quality of Life (QOL) from the internationally validated Quality of Life Enjoyment and Satisfaction Questionnaire [26]; Food Insecurity from The Household Food Insecurity Access Scale (HFIAS); [27], that measures food insecurity in India and elsewhere [28,29,30]; Depression from The Center for Epidemiological Studies Depression Scale (CES-D), short version, a 10-item depression scale with well-established reliability and validity [31] including in India [32]; Internalized stigma, a 10-item scale measuring the extent that respondents believe they deserved to be shunned (five items, e.g., should avoid social interactions) or blamed/shamed because of HIV (five items, e.g., shamed the family, feel guilty) studied in Indian pop [25,33,34]; Social Support from The MOS Social Support Survey [35]; and Adherence to ART, a self-reported measure that assessed adherence to ART via a Visual Analogue Scale ranging from 0 to 100, best representing the percentage of pills taken in past month [22,36].

Biological measures included: months living with HIV/AIDS, derived from asking subjects about month and year of HIV diagnosis; CD4+ T cell count from blood samples collected at screening with CD4+ T cell count determination by flow cytometry at the district hospital lab. Body composition was measured at baseline and at three months, six months, twelve months and eighteen months by trained research staff who used a calibrated scale to weigh WLA while barefoot, with heights measured using a stadiometer. A portable Bioimpedance Analyzer (model 310e, Biodynamics, Inc, Seattle, WA, USA) measured percent body fat, fat mass and lean mass based on our protocol developed during our pilot investigation [9]. Body mass index (BMI) was computed as weight in kilograms divided by height in meters squared. We constructed BMI categories using the World Health Organization (WHO) International Criteria for all populations (<18.5 kg/m^2^; 18.5–24.9 kg/m^2^; 25–29.9 kg/m^2^; >30 kg/m^2^), and the WHO International Criteria for Asian populations (<18.5 kg/m^2^; 18.5–22.9 kg/m^2^; 23–27.49 kg/m^2^; >27.5 kg/m^2^) [37].

Physical Activity collected from International Physical Activity Questionnaire (IPAQ) was summarized into metabolic equivalent of energy expenditure (MET) minutes per week [38]. Opportunistic infections (OI) were assessed by using a list of eight common OIs endorsed by our collaborating physicians (e.g., tuberculosis, fungal dermatoses) and participants indicating whether they had experienced OI in the previous six months, with a summation of OI into one index measure.

Dietary Assessment. We conducted assessment of underlying dietary consumption using the NINA-Dish, a nutritional assessment tool designed by Indian Investigators to measure foods found in regions of India (New Delhi, Mumbai, Kerala and Trivandrum) that did not include Nellore [39,40,41]. Because of the sizeable variation in Indian cuisine [42], we modified the NINA-DISH to accurately record our participants’ diet by identifying foods unique to the Nellore region and inserting the unique foods and associated nutrients into the NINA-DISH software. A tablet-based platform was developed to house the NINA-DISH software. The NINA-DISH software was validated using a small baseline sub-sample of Indian women who were newly recruited to the cluster randomized trial of protein supplementation prior to start-up. The validated NINA-DISH tablet assessed the nutritional status of all 600 participants at multiple time points during intervention and follow-up periods that included baseline, monthly intervals from month one to month six during the intervention period, and at both 12 and 18-months during follow-up. Results from the dietary assessment of the baseline study population will be reported in a future publication.

### 2.6. Statistical Methods

#### 2.6.1. Sample Size Estimation

Sample size calculations that informed establishment of intervention group size were based on preliminary results from our earlier conducted behavioral and nutritional pilot intervention trial [9]. To ensure balance in HIV prevalence between the four geographic areas, we randomly selected 20 primary health centers from the 72 PHCs that occur in the Nellore region and organized the geographic regions to represent an even distribution of centers. In addition, the four areas represented contiguous villages that were clustered within an area yet geographically distinct from the other intervention areas. To evaluate power, we assumed an intraclass correlation of 0.05 to account for potential clustering induced by the grouping of participants in the geographic intervention areas. A sample of 600 WLA was anticipated to provide 30 WLA per PHC with a design effect of 2.45. We also conservatively set α = 0.01 to mitigate for the fact that we had more than one outcome to test (rather than apply the overly conservative Bonferroni correction for multiple testing). Under these assumptions, we had 80% power to detect, for each main effect stated, differences of about half a standard deviation (SD) in size (d = 0.48) between the mean of the groups receiving food or education vs. the groups not receiving this intervention factor.

#### 2.6.2. Data Collection

Interview data collected in the field from Android^®^ tablets by the interviewers were uploaded using cell phone signal or wi-fi from our study sites in Andhra Pradesh to a Microsoft server located on the UCLA campus with data resident on the UCLA server available in an EXCEL-format identified by numeric participant ID.

#### 2.6.3. Data Analysis

All data analyses were conducted using SAS 9.4^®^ (Statistical Analysis System, Cary, NC, USA). One-way ANOVA models (for continuous variables) and Chi-Square tests (for categorical variables) assessed baseline differences according to intervention group. Variables that differed by treatment group (*p* < 0.10) at baseline were included as covariates in subsequent longitudinal models. We assessed whether covariates that were significantly different at baseline might affect outcome according to intervention condition by adjusting for each individual significant covariate and for all covariates together using mixed modeling. Maximum likelihood estimation was used to assess relative contribution of covariates to the mixed models that assessed impact of interventions on the primary and secondary outcomes. The main models evaluated between group differences modeled within the construct of within subjects’ repeated measurements at the time periods of six, twelve and 18 months. The repeated terms were evaluated within the mixed model where fixed terms represented the intervention group effects, time, group by time and the covariates that differed at baseline. Because random allocation was conducted at the group level and analyses conducted at the individual level, the two-level model allowed for clustering to occur at the individual level within groups. To determine whether clustering may have impacted our estimates, we fit our models with a random term for individual clustering within groups and compared model fit with and without the random term.

For the primary (lean muscle mass) and secondary outcomes (fat mass and weight), we used repeated measures to evaluate changes from baseline to the time points (6, 12, and 18 months). Within the mixed model specifications, we built contrasts using indicator variables to represent the factorial design with outcome means for each factorial condition compared to usual care control. We employed a compound symmetry mixed model that utilized maximum likelihood estimation to determine change in model fit based on covariate inclusion.

Crude mixed models assessed the impact of factorial conditions on change since baseline observed in the outcome variables. To account for the potential influence of differences at baseline according to the intervention group, final models were fully adjusted for covariates (age, exercise, quality of life, food insecurity, social support, internalized stigma, education and adherence). Models were additionally stratified at the median cut-point for adherence to ART medication since adherence influences nutritional status and body composition [14]. An interaction product term between adherence and treatment × time was constructed and model fit was evaluated with and without addition of the interaction term. Significance was indicated by *p*-values less than 0.05. Further details describing the statistical modeling approach can also be found in our previous investigations [20,21].

## 3. Results

### 3.1. Overall Study Sample

Table 1 and Table 2 describe the frequency distributions of continuous (Table 1) and categorical variables (Table 2) at baseline for the total study population and population in the four intervention groups. In the overall study population, participants were 34 years old on average; almost half of the population (49%) were lacking an education. Participants experienced an average of 4.6 opportunistic infections over the past six months. Participants averaged 46 kg in weight with a BMI of 20 kg/m^2^, with 34 kg lean mass and 12 kg fat mass.

### 3.2. Study Sample by Group

Table 1 contains results from ANOVA models of continuous covariate means evaluated according to intervention group at baseline. Since randomization was not performed at the individual level, some group differences were expected. Age, QOL, summary social support, MET, internalized stigma and education were significantly different at baseline according to group, and included as covariates in subsequent models. Percent adherence to ART medication was not statistically significant (*p* = 0.18) at baseline, but was retained as a covariate given its clinical importance in relationship to the outcomes [14].

Two-by-two cross-tabulations evaluated categorical variable distributions at baseline according to intervention (see Table 2). Deviations between observed and expected frequencies were assessed using Chi-Square tests. One categorical variable, years of education (*p* = 0.04), had a *p*-value less than 0.10 and was included as a covariate along with the continuous covariates in subsequent adjusted models.

### 3.3. Anthropometric Outcomes

Crude mixed models were constructed with lean mass at baseline and follow-up as the primary outcome, and fat weight and overall weight as secondary outcome variables (see Table 3). We reported effects from total weight at baseline and six months in an earlier publication [20], but are including results in this publication because we extended the follow-up period beyond six months to 12 and 18 months, and the model required including all time points. We used mixed modeling with repeated measures and factorial constructs to evaluate lean mass effects across the time periods compared to baseline according to treatment group. We determined whether clustering impacted our estimates by fitting our models with a random term for individual clustering within groups and compared model fit with and without the random term. Model fit was not appreciably different. Table 3 reports results from fully adjusted models. Lean mass change was significantly and independently associated with nutrition education (*p* = 0.0001) and nutrition supplementation (*p* = 0.0001) at six months, and these effects were sustained at 12 months and 18 months. Combined nutrition supplementation and education was significantly associated with change in lean mass (see Table 3) across the three time points. 

The secondary outcomes, changes in fat mass and body weight since baseline, were also evaluated using mixed models with the same factorial structure. Nutrition supplementation was associated with increasing fat mass (adjusted *p* < 0.05) at all time points, while nutrition education was not associated with any of the time points (18 months *p* = 0.6089). Combined nutrition supplementation and education was associated with increasing fat mass at all time points (6, 12, and 18 months (*p* = 0.0001). Both nutrition supplementation (*p* = 0.0001), and nutrition education (*p* = 0.0007) were strongly and independently associated with changes in overall weight at 12 and 18 months. Nutritional supplementation was associated at six months (*p* = 0.0001), but nutrition education was not (*p* = 0.2670). Combined nutrition supplementation and education was not significantly associated with changes in overall weight at six months (*p* = 0.83), but was associated with changes in overall weight at 12 months (*p* = 0.0003) and 18 months (*p* = 0.0001) (see Table 3).

### 3.4. Anthropometric Outcomes: Stratified by Adherence to ART

We assessed associations with the primary and secondary outcomes stratifying by above and below the median of adherence and found relative consistency of effects for lean mass and fat mass (see Table 4). Among individuals who were poorly adherent (adherence < 30%), we observed significant associations between nutritional supplementation and change in lean mass and change in overall weight, but not fat mass. Nutrition education was also associated with improvement in lean mass and overall weight outcomes, but not fat mass. The combined nutrition supplementation and education was associated with primary and secondary outcomes. Among participants who had better adherence (adherence > 30%), nutrition education was significantly associated with improvement in lean mass and overall weight outcomes, while nutrition supplementation was associated with all outcomes. Combined nutrition supplementation and education were associated with improvement in all body composition outcomes. Overall effect modification between adherence and associations between the interventions and changes in body composition was not apparent (*p* > 0.05) for lean mass and fat mass but was apparent for overall weight (*p* = 0.0239). 

## 4. Discussion

We report results from a cluster-randomized-factorial-nutrition-controlled trial with a six-month intervention period, followed by 12 month and 18-month follow-up periods. Compared to an intervention arm which included community-based Asha support alone, we evaluated relative contributions of nutrition education, nutritional supplementation and combined nutrition education and supplementation on changes in lean mass, fat mass, and weight from baseline to the three follow-up periods. Our results showed a strong independent effect of nutrition education and a strong independent effect of nutrition supplementation on gains in lean mass six-months after baseline that was sustained at 12 months and 18 months. Combined nutrition education and supplementation was additionally associated with improved lean mass across all follow-up periods. Weight gain showed similar patterns, although for nutrition education the effect was shown in the later time periods, at 12 months and 18 months. Weight gain was significantly associated with nutrition supplementation alone across all time periods. Nutrition supplementation combined with nutrition education was also associated in the later time periods (12 and 18 months). Fat mass showed similar patterns with effects seen in the later time periods. These associations were further stratified according to percent adherence to ART medication. Association with the outcomes and interventions showed some differences according to adherence with total weight more strongly associated with the intervention conditions among participants who were more adherent to ART. Associations with lean mass and fat mass were not significantly different according to adherence. Study results point to important influences of nutrition both in terms of supplementation as well as education, and when provided in conjunction with an AIDS self-management intervention inclusive of Asha support result in improved lean mass as well as body weight and fat mass among women living with HIV/AIDS in rural South India.

Interventions that target nutrition may be an important approach to improving physiologic response to HIV. Muscle wasting or the breakdown of muscle tissue through catabolism into amino acids often signifies a worsening of disease. Not only does HIV pathophysiology promote muscle catabolism, but anti-retroviral therapies can affect mitochondrial function and raise the resting metabolic rate [43]. The increased metabolic load combined with living in impoverished conditions can lead to a vicious cycle which serves to hasten mortality. The concurrent burden of infectious diseases that are both opportunistic and endemic in the same region can further weaken the immune response and augment malnutrition and cachexia [44]. Gain of lean mass resulting from protein supplementation and nutrition education observed in our study may, because of links to immune function, improve immune response to HIV. Our previous investigations noted the improvement in CD4 count resulting from the interventions [20,21], and our current finding of improved lean mass is consistent with reports in the literature linking lean mass increases with improved immune response [7]. 

**Figure 2:**
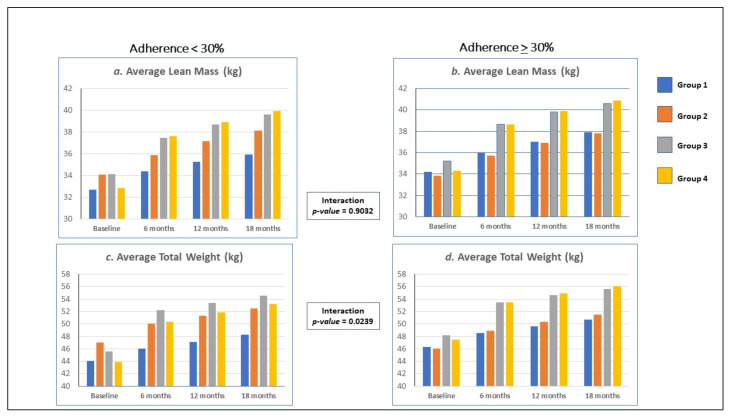
Group by Time Means of Lean Mass and Total Weight by Adherence. (**a**) Adherence < 30%, Lean Mass by Group by Time; (**b**) Adherence > 30%, Lean Mass by Group by Time; (**c**) Adherence < 30%, Total Weight by Group by Time; (**d**) Adherence > 30%, Total Weight by Group by Time.

Body composition improvements have been observed with ART therapy [14,45], and our observed gain in lean mass could have partially resulted from improved ART adherence and not the nutrition interventions. Percent adherence at baseline was not significantly different between the intervention conditions at baseline (*p* = 0,18), but because of the importance of adherence as a covariate, we adjusted for adherence in our mixed models of intervention effects. We presented intervention effects stratified by median percent adherence. We observed relatively consistent associations with lean mass and fat mass but not total weight (see Table 4 and Figure 2). Our earlier publication studied total weight effects at six months compared to baseline stratified by adherence, but associations were more uniform across adherence [20]. Observable interaction between adherence, total weight and the intervention conditions suggest that adherence in combination with the nutritional interventions exceed multiplicative effects on total weight (*p* = 0.0239). We further evaluated mixed models of body mass index, whose effects were reported in our previous publications [20,21], by adherence and similarly found observable interaction with adherence (*p* = 0.0028). We could potentially have modification of lean mass and fat mass, however parceling out body composition into lean mass and fat mass may compromise the power to detect interactive effects. 

Cultural practices in India may influence dietary consumption in general, and these consumption patterns may influence how well residents meet the metabolic demands of contracting HIV. India has one of highest proportions of vegetarians in the world. According to a report from the third India’s National Family Health Survey, 36% of the surveyed population practices some form of vegetarianism [46]. In addition, up to 60 to 70% of the diets in the Indian population are represented by carbohydrate consumption [47], with individuals living in South India consuming up to 66% carbohydrates in their diets. While no studies have directly examined whether potential dietary protein deficiencies found among vegetarians may put them at higher risk for mortality from HIV, these studies suggest that the high prevalence of vegetarianism and strain of HIV on endogenous protein stores in muscle may put individuals living with HIV/AIDS in India at an even higher risk for mortality than individuals living in other countries where protein is more available.

Our nutritional intervention targeted supplementation of participants’ diets with additional protein and calories represented by indigenous food consistent with dietary preferences of residents in rural Andhra Pradesh. Our previously conducted pilot investigation demonstrated palatability and uptake of the dietary intervention [9], and we chose the same grain provider in Kovur that we used previously. Our dietary protein supplementation relied on high protein dal lentils that were consistently measured and provided to participants’ entire families due to the cultural practice of Indian women eating their meals after rest of the family ate. We observed consistent increases in lean mass across all time periods with nutritional supplementation, and with nutritional supplementation combined with nutritional education. 

We found an independent association between individuals who participated in our nutrition education intervention and increase in lean mass across all time points, but not fat mass. Our educational efforts were targeted toward deficiencies that are likely to impact HIV prognosis, but did not address calorie deficits. Fat mass did not increase but an overall weight increase was observed in the later follow up periods (12 months and 18 months). The association between nutrition education and overall weight observed in the later follow-up periods suggest that nutrition education effects may take longer to occur and may be potentially more sustainable than nutritional supplementation. 

There are several limitations to our study. We used BIA to measure lean mass and fat mass. Lean mass, or fat-free mass measured by the BIA, is only an approximation of muscle mass and measurements of lean body mass in populations can be highly variable, as changes in hydration status increases variability [48]. The challenging circumstances faced by our study population did not permit the administration of more refined anthropometric measurements such as DXA or CT scanning. In our investigations of body composition using BIA at the UCLA Center for Human Nutrition, we found the BIA to be a reasonable approximation of lean and fat mass [49], and when compared to BMI in multi-ethnic groups, it was a better approximation of body composition than BMI alone [50]. 

Our study design may have led to the confounding of observed associations by other factors. The random allocation of intervention conditions was not conducted on an individual level, and unmeasured factors may not have been fully adjusted for in the analysis. Moreover, there may have been inherent differences due to the variation in geographic location of the clusters with potential for cross-contamination of intervention conditions. Road conditions and lack of vehicular travel greatly limited mobility of the study populations between the study sites. Visits to the centralized study office for intervention delivery were scheduled on different days for each study condition and the opportunity for loss of intervention fidelity was minimized. We further accounted for potential underlying clustering in our mixed models by constructing a random effects term for within group clustering on the individual level and found no appreciable impact on our model estimates. 

Our study results may have been influenced by recall bias because we relied on self-reported month and year of HIV diagnosis. Our onsite study manager had access to health records located at the PHC Centers and the district hospital. She performed ‘spot checking’ of diagnosis date with responses to study questions. We found consistency with recall of HIV diagnosis and information recorded in the records, although we did not check every record. Bias is unlikely to be differential, however, since participants’ knowledge about potential impact of time since diagnosis on study results was not evident.

## 5. Conclusions

Our findings conclusively demonstrate the efficacy of including nutrition supplementation and nutrition education both independently and in combination to improve body composition of women living with HIV/AIDS in rural South India. We observed immediate improvement at six months as well as sustained improvement up to 18 months for lean mass, fat mass, and total weight. Stratification of observed associations by ART adherence demonstrated consistency of association according to adherence for lean mass and fat mass with interaction occurring between adherence, total weight and the nutritional interventions. Stronger effects for total weight were observed among women who had greater adherence to their ART medication.

## Figures and Tables

**Table 1:** Description at Baseline of Total Study Population According to Group Membership.

Continuous Variables	Total Study Population		ASHA (AS) Control		Nutr. Education + AS		Nutrition + AS		Nutr. Educ + AS		
	(*n* = 600)		(*n* = 150)		(*n* = 150)		(*n* = 150)		(*n* = 150)		F-Test
Variable	Mean	Std Dev	Mean	Std Dev	Mean	Std Dev	Mean	Std Dev	Mean	Std Dev	*p*-Value ^1^
Age	34.30	6.97	36.05	6.98	34.66	6.91	33.6	6.42	32.91	7.18	0.001
Number of Children	1.86	0.80	1.83	0.73	1.78	0.78	1.91	0.93	1.92	0.76	0.37
Monthly Income (INR)	2113.50	678.68	2110.00	758.9	2158.67	611.98	2137.33	626.97	2048.00	707.93	0.52
Quality of Life	0.30	0.29	0.26	0.31	0.34	0.25	0.27	0.33	0.33	0.24	0.03
Percent Adherence	30.37	13.23	30.37	12.75	32.00	14.35	28.60	12.99	30.50	12.69	0.17
Number of OI past 6 months	4.58	1.21	4.61	1.35	4.52	1.35	4.58	0.96	4.63	1.13	0.88
Time since HIV diagnosis (Year)	4.05	2.88	4.34	3.25	3.83	2.44	4.10	2.82	3.95	2.96	0.46
Internalized Stigma	2.30	0.25	2.24	0.41	2.30	0.16	2.33	0.19	2.32	0.15	0.02
Summary CESD Depression	9.18	3.08	9.12	3.11	9.09	3.06	9.16	3.13	9.35	3.04	0.88
Summary Social Support	1.08	0.22	1.13	0.41	1.06	0.12	1.06	0.08	1.06	0.08	0.004
Summary Food Insecurity	21.14	3.44	20.27	4.29	21.63	2.58	20.76	3.45	21.94	2.98	0.001
Total MET ^2^ min/week	4608.57	1663.57	4957.09	1873.23	4569.73	1416.82	4691.85	1718.60	4215.60	1539.27	0.001
Body Mass Index kg/m^2^	20.10	4.17	19.81	3.78	20.20	3.97	20.55	4.67	19.86	4.16	0.38
Weight (kg)	46.25	10.37	45.30	9.66	46.42	9.65	47.43	11.22	45.84	10.86	0.33
Height (cm)	151.52	5.98	151.06	5.81	151.57	6.06	151.85	5.80	151.62	6.27	0.71
Lean Muscle Mass (kg)	33.96	6.89	33.53	6.62	33.95	6.69	34.70	7.11	33.66	7.11	0.46
Fat Mass (kg)	12.25	5.75	11.62	5.07	12.48	5.33	12.73	6.36	12.19	6.15	0.38

^1^ Evaluation of between group differences at baseline using one-way ANOVA; ^2^ Metabolic Equivalent of Energy Expenditure; *p*-value < 0.10; Percent Adherence included as covariate.

**Table 2:** Description at Baseline of the Total Population and Population According to Group Membership.

Categorical Variables		Total Study Population		Asha (AS) Control		Nut. Educ + AS		Nut Supp + AS		Nut Educ + Supp+ AS		Chi
		(*n* = 600)		(*n* = 150)		(*n* = 150)		(*n* = 150)		(*n* = 150)		Square
Variable	Category	*N*	%	*N*	%	*N*	%	*N*	%	*N*	%	*p*-Value ^1^
Marital	married	238	39.67	51	34.00	67	44.67	53	35.33	67	44.67	
Status	divorced	54	9.00	12	8.00	15	10.00	12	8.00	15	10.00	
	widowed	308	51.33	87	58.00	68	45.33	85	56.67	68	45.33	0.19
Education	None	292	48.67	85	56.67	74	49.33	71	47.33	62	41.33	
	<5 years	98	16.33	25	16.67	29	19.33	25	16.67	19	12.67	
	5–9 years	123	20.50	24	16.00	31	20.67	33	22.00	35	23.33	
	>10 years	87	14.50	16	10.67	16	10.67	21	14.00	34	22.67	0.04
Religion	Hindu	439	73.17	110	73.33	116	77.33	104	69.33	109	72.67	
	Muslim	44	7.33	17	11.33	5	3.33	12	8.00	10	6.67	
	Christian	117	19.50	23	15.33	29	19.33	34	22.67	31	20.67	0.14
Body	<18.5 kg/m^2^	234	39.00	59	39.33	57	38.00	55	36.67	63	42.00	
Mass	18.5–22.9	228	38.00	63	42.00	54	36.00	57	38.00	54	36.00	
Index	23–27.49	103	17.17	24	16.00	30	20.00	23	15.33	26	17.33	
WHO-Asian	27.5+	35	5.83	4	2.67	9	6.00	15	10.00	7	4.67	0.35
Body	<18.5 kg/m^2^	234	39.00	59	39.33	57	38.00	55	36.67	63	42.00	
Mass	18.5–24.99	281	46.83	75	50.00	73	48.67	69	46.00	64	42.67	
Index	25.0–29.99	69	11.50	13	8.67	16	10.67	20	13.33	20	13.33	
WHO-Int	30+	16	2.67	3	2.00	4	2.67	5	3.33	4	2.69	0.61

^1^ Evaluation of between group differences using tabular frequencies and Chi Square Distribution. *p*-value < 0.10.

**Table 3:** Group Means and Mixed Model Effects for Primary and Secondary Outcomes ^1^.

Primary Outcome: Lean Mass										
	Group Means								Time Effects	
	Asha (AS)		Nut Educ		Nut Supp.		Nut Educ +		Time Effects	
	Control		+ AS		+ AS		Nut Supp. + AS		(within Subjects)	
	Mean	Std Dev	Mean	Std Dev	Mean	Std Dev	Mean	Std Dev		*p*-Value
Baseline Lean Mass	33.52	6.62	33.95	6.70	34.70	7.11	33.66	7.12		
6 Month Lean Mass	35.28	6.24	35.78	6.75	38.10	7.16	38.17	6.82	4.52	0.001
12 month Lean Mass	36.25	6.23	36.99	6.58	39.29	7.05	39.45	6.62	5.79	0.001
18 month Lean Mass	37.03	6.28	37.92	6.60	40.13	7.05	40.45	6.60	6.79	0.000
	Group and Time Effects									
		*p*-value		*p*-value		*p*-value		*p*-value		
Group Effects (between subjects)										
			21.73	0.001	20.86	0.001	20.33	0.001		
Time by Group:										
6 months			−1.11	0.001	−2.68	0.001	−2.76	0.001		
12 months			−1.20	0.001	−2.75	0.001	−3.06	0.001		
18 months			−1.36	0.001	−2.82	0.001	−3.28	0.001		
Type 3 Tests of Fixed Effects	F-value	*p*-value								
Time	1342.45	0.001								
Group	6.31	0.003								
Time by Group	37.00	0.001								
Overall Maximum Likelihood										
Ratio Test for Adjusted Model										
DF	Chi Sq	*p*-value								
7	4721.28	0.001								
Secondary Outcome: Fat Mass										
	Group Means								Time Effects	
	Asha (AS)		Nut Educ		Nut Supp.		Nut Educ +		Time Effects	
	Control		+AS		+AS		Nut Educ + AS		(within subjects)	
	Mean	Std Dev	Mean	Std Dev	Mean	Std Dev	Mean	Std Dev		*p*-value
Baseline Fat Mass	11.62	5.07	12.48	5.33	12.73	6.36	12.19	6.15		
6 month Fat Mass	12.19	5.09	13.55	5.28	14.79	6.89	13.91	5.89	1.72	0.0001
12 month Fat Mass	12.29	4.86	13.71	5.11	14.76	6.56	13.97	5.85	1.78	0.0001
18 month Fat Mass	12.62	4.78	13.96	4.99	15.01	6.55	14.35	5.74	2.16	0.0001
	Group and Time Effects									
		*p*-value		*p*-value		*p*-value		*p*-value		
Group Effects (between subjects)			2.01	0.56	2.36	0.53	1.83	0.6764		
Time by Group:										
6 months			0.34	0.14	−0.64	0.002	−1.15	0.001		
12 months			0.25	0.28	−0.54	0.010	−1.11	0.001		
18 months			0.12	0.61	−0.67	0.001	−1.15	0.001		
Type 3 Tests of Fixed Effects	F-value	*p*-value								
Time	204.47	0.001								
Group	5.36	0.001								
Time by Group	8.44	0.001								
Overall Maximum Likelihood										
Ratio Test for Adjusted Model										
DF	Chi Sq	*p*-value								
7	4395.62	0.001								
Secondary Outcome: Weight (kg)										
	Group Means								Time Effects	
	Asha (AS)		Nut Educ		Nut Supp.		Nut Educ +		Time Effects	
	Control		+ AS		+ AS		Nut Supp. + AS		(within subjects)	
	Mean	Std Dev	Mean	Std Dev	Mean	Std Dev	Mean	Std Dev		*p*-value
Baseline Weight (kg)	45.30	9.66	46.42	9.65	47.43	11.22	45.84	10.86		
6 Month Weight	47.47	9.56	49.33	9.63	52.89	11.02	52.08	10.74	6.24	0.001
12 month Weight	48.54	9.51	50.70	9.56	54.06	11.02	53.56	10.70	7.72	0.001
18 month Weight	49.66	9.46	51.88	9.52	55.14	10.99	54.80	10.61	6.24	0.001
	Group and Time Effects									
		*p*-value		*p*-value		*p*-value		*p*-value		
Group Effects (between subjects)			22.21	0.002	23.38	0.001	21.82	0.0021		
Time by Group:										
6 months			−0.78	0.001	−3.33	0.001	−4.06	0.001		
12 months			−1.09	0.001	−3.44	0.001	−4.48	0.003		
18 months			−1.24	0.001	−3.50	0.001	−4.60	0.001		
Type 3 Tests of Fixed Effects	F-value	*p*-value								
Time	8127.26	0.001								
Group	7.69	0.003								
Time by Group	209.08	0.001								
Overall Maximum Likelihood										
Ratio Test for Adjusted Model										
DF	Chi Sq	*p*-value								
7	8237.26	0.001								

^1^ Adjusted for all covariates significantly different at baseline (age, total MET-minutes, Quality of Life, food insecurity, social support, internalized stigma, years of education, percent adherence). ^2^ Assessed by adding product term of adherence by group by time to fully adjusted model.

**Table 4:** Mixed Model Effects of Interventions by Adherence.

	Adherence < 30% (*n* = 258)						Adherence > 30% (*n* = 342)						
	Intervention Group by Time Effects						Intervention Group by Time Effects						
	Nut Educ		Nut Supp.		Nut Educ & Supp		Nut Educ.		Nut Supp.		Nut Educ & Supp		Interaction
		*p*-Value ^1^		*p*-Value		*p*-Value		*p*-Value		*p*-Value		*p*-Value	*p*-Value ^2^
Primary Outcome													
Lean Mass													
6 months	−1.42	0.0001	−2.98	0.0001	−3.07	0.0001	−0.86	0.0210	−2.45	0.0001	−2.51	0.0001	
12 months	−1.46	0.0001	−2.99	0.0001	−3.48	0.0001	−0.99	0.0080	−2.56	0.0001	−2.73	0.0001	
18 months	−1.60	0.0001	−3.07	0.0001	−3.84	0.0001	−1.17	0.0017	−2.61	0.0001	−2.84	0.0001	0.9032
Secondary Outcomes													
Fat Mass													
6 months	0.59	0.0751	−0.50	0.1779	−1.12	0.0018	0.12	0.7132	−0.73	0.0029	−1.67	0.0001	
12 months	0.45	0.1690	−0.57	0.1275	−0.97	0.0068	0.07	0.8225	−0.52	0.0355	−1.22	0.0001	
18 months	0.29	0.3850	−0.75	0.0447	−0.93	0.0097	−0.03	0.9195	−0.62	0.0112	−1.34	0.0001	0.5491
Weight (kg)													
6 months	−0.84	0.0001	−3.49	0.0001	−4.50	0.0001	−0.74	0.0001	−3.18	0.0001	−3.72	0.0001	
12 months	−1.13	0.0001	−3.69	0.0001	−4.89	0.0001	−1.07	0.0001	−3.23	0.0001	−4.15	0.0001	
18 months	−1.31	0.0001	−3.82	0.0001	−5.08	0.0001	−1.21	0.0001	−3.22	0.0001	−4.22	0.0001	0.0239

^1^ Adjusted for all covariates significantly different at baseline (age, total MET-minutes, Quality of Life, food insecurity, social support, internalized stigma, years of education, percent adherence). ^2^ Assessed by adding product term of adherence by group by time to fully adjusted model.

## Data Availability

Data are available upon request.
